# Cardiac hypertrophy is stimulated by altered training intensity and correlates with autophagy modulation in male Wistar rats

**DOI:** 10.1186/s13102-019-0121-0

**Published:** 2019-06-10

**Authors:** Julia Windi Gunadi, Vita Murniati Tarawan, Iwan Setiawan, Ronny Lesmana, Roro Wahyudianingsih, Unang Supratman

**Affiliations:** 1 Department of Physiology, Faculty of Medicine, Maranatha Christian University, Bandung, Indonesia; 2 Physiology Division, Department of Biomedical Science, Faculty of Medicine, Universitas Padjadjaran, Bandung, Indonesia; 3 Division of Biological Activity, Central Laboratory, Universitas Padjadjaran, Bandung, Indonesia; 4 Center of Excellence in Higher Education for Pharmaceutical Care Innovation, Universitas Padjadjaran, Bandung, Indonesia; 5 Department of Anatomy Pathology, Faculty of Medicine, Maranatha Christian University, Bandung, Indonesia; 6 Department of Chemistry, Faculty of Mathematics and Natural Sciences, Universitas Padjadjaran, Bandung, Indonesia

**Keywords:** Autophagy, Training, Cardiac hypertrophy, αMHC, PIK3CA, mTOR, LC3, p62

## Abstract

**Background:**

The mechanism for cardiac hypertrophy process that would be a benefit for improvement of cardiovascular endurance needed to be investigated throughly. Specific intensity of training may play a role for homeostasis process in cardiac during training. In the present study, we examine the effect of different intensity of treadmill training on cardiac hypertrophy process and autophagy related gene expression in male wistar rats.

**Methods:**

Three different intensities of treadmill training were conducted on 15 male wistar rats (Low Intensity: 10 m/minute, Moderate Intensity: 20 m/minute, and High Intensity: 30 m/minute) compared to 5 sedentary rats as control. Training duration was 30 min per day, frequency was 5 days per week, during 8 weeks period. Heart weight and heart weight/body weight ratio were measured after the experiments. Left ventricle myocardium was taken for microscopic analysis with HE staining. mRNA was extracted from left ventricle myocardium for examining αMHC and autophagy related gene expression (PIK3CA, mTOR, LC3, p62) using semi quantitative PCR.

**Results:**

We observed that altered training intensity might stimulate cardiac hypertrophy process. MI and HI training increased heart weight and heart weight/body weight ratio. This finding is supported by microscopic result in which cardiac hypertrophy was found in MI and HI, with focal fibrosis in HI, and increased αMHC gene expression in MI (*p* < 0.05) and HI (*p* = 0.076). We also observed decreased PIK3CA (LI 0.8 fold, MI 0.9 fold), mTOR (LI 0.9 fold, MI 0.9 fold), LC3 (LI 0.9 fold, MI 0.8 fold, HI 0.8 fold), and p62 (LI 0.8 fold, MI 0.9 fold) compared to control. Interestingly, we found increased mTOR (HI 1.1 fold) and p62 (HI 1.1 fold) compared to control.

**Conclusion:**

Training with different intensity creates different cardiac hypertrophy process based on heart weight and heart weight/body weight ratio, microscopic examination and autophagy related gene expression.

**Electronic supplementary material:**

The online version of this article (10.1186/s13102-019-0121-0) contains supplementary material, which is available to authorized users.

## Background

Cardiovascular fitness can be improved by regular training. A well-trained athlete can achieve a cardiac output double that of a sedentary person, in part because training causes cardiac hypertrophy, which is defined as enlargement of the heart [1]. Training stimulates increase of cardiac performance, which is initiated by anatomical tissue rearrangement, followed by optimizing its function, called as physiological cardiac hypertrophy. In the other side, pathological cardiac hypertrophy indicated by anatomical change like fiber replacements, loss of cardiomyocytes, lead to heart failure and sudden death [2, 3].

Cardiac hypertrophy is initiated in order to follow process of physiology. Physiological cardiac hypertrophy can be defined as a benign increase in heart mass with morphological alteration, which represents a physiological adaptation to chronic training. There has been some questions about whether high intensity training could develop pathological cardiac hypertrophy, but there is no evidence showing remodeling due to training leads to long-term cardiac disease progression, cardiovascular disability, or sudden cardiac death [4, 5]. Furthermore, left ventricle hypertrophy after long term training is reversible following detraining [6, 7], so it can be concluded that physiological cardiac hypertrophy induced by training is a benign adaptation [2].

Sustained training increased the oxygen demand of the muscles. Whether the demand is met depends mainly on the adequacy of cardiac output and proper functioning of the respiratory system. After several weeks of training, cardiac output is increased, which also increases the maximal rate of oxygen delivery to tissues/VO_2_max [1]. Many studies confirmed that cardiac adaptations to training are closely related to increased VO_2_max. However, little is known about cardiac hypertrophy related to different intensity, especially at molecular level. The question remains about how much training is optimal for cardiovascular benefit and what molecular mechanism for cardiac hypertrophy process that would be a benefit for improvement of cardiovascular endurance [8, 9].

Genetic mouse models have provided substantial evidence about molecular pathway that regulates physiological cardiac growth. Signaling cascades responsible for mediating physiological cardiac hypertrophy is IGF1-phosphoinositide 3-kinase (PI3KCA/p110α)-Akt-mTOR pathway [2, 4]. mTOR is an atypical serine/threonine protein kinase that affects gene transcription, protein translation, regulation of cell growth, apoptosis, and autophagy [10]. mTOR is encoded by a single gene in mammals and represents the catalytic subunit of mTORC1, which is the main regulator of cellular growth in response to different environmental and intracellular conditions. It promotes anabolic process such as protein synthesis while inhibits catabolic pathways such as autophagy in cardiovascular system [11, 12] .

Autophagy is a conserved mammalian catabolic process by which unwanted cellular cargos and dysfunctional organelles are discarded in a lysosome-dependent manner [13]. Autophagy in cardiovascular system can be referred as cardiac autophagy. Endurance training may alter cardiac autophagy that leads to a protective role against hypoxia and ischemia-reperfusion injury [14, 15]. Recent studies have shown that acute and chronic endurance training enhances autophagy in cardiac muscles [16–19]. A proper regulation of cardiac autophagy is important because a chronic upregulation of cardiac autophagy may induce a detrimental effect.

In cardiac hypertrophy, cardiac myocytes rearrangement by different intensity of chronic endurance training may strongly involves autophagy. However, the effect of different intensity of chronic training on cardiac autophagy remains unclear. The objective of this study is to examine the effect of different intensity of treadmill training on cardiac hypertrophy process and autophagy related gene expression in male Wistar rats. In the present study, we postulate that different intensity of training creates a different response in cardiac hypertrophy and correlates with cardiac autophagy.

## Methods

### Animals

We obtained 20 male wistar rats aged 8 weeks, weighed 200 ± 50 g, from PT. Biofarma, Bandung, Indonesia. The rats were placed in a standardized cage (5 rats in each cage), given pellet rodent diet (normal Chow Diet) and water ad libitum every day. The dark and light cycle environment were maintained within 12 h with stable humidity and temperature around ±22–24 °C. Adaptation to environment conducted in 2 weeks period, food and water provided ad libitum. The procedures for treatment of the animals were conducted according to the guide for the use and care of laboratory animals [20] and were approved by Research Ethics Committee of Universitas Padjadjaran with approval number, No 676/UN6.KEP/EC/2018.

### Treadmill training protocol

Twenty male-wistar rats were divided into four groups: 3 groups of treadmill training (Low Intensity with treadmill speed 10 m/minute; Moderate Intensity with treadmill speed 20 m/minute, and High Intensity with treadmill speed 30 m/minute) and sedentary control (*n* = 5 for each group). This number of rats for each groups was calculated based on minimal sample calculation. The treadmill training intensities were defined based on lactate accumulation levels and followed previous study [21]. Rats were randomly allocated to 4 groups upon arrival, then acclimatized and habituated to environment for 2 weeks, followed by treadmill adaptation for another 2 weeks, then continued with treadmill training for 8 weeks, with frequency 5 days per week, and duration 30 min per day (Additional file 1: Figure S1). This study followed the protocol described by Vita et al., with a different detail purposes and scheme of the research [22].

At last training day, on 9 o’clock in the morning, in Animal Laboratorium of Universitas Padjadjaran, rats were sacrificed immediately after last training under inhaled isoflurane flow rate or concentration to 5% or greater, continued until 1 minute after breathing stopped [23]. Isoflurane was chosen as the anaesthetic drugs according to ethical approval as issued. Heart samples were collected; weighed; and dissected to separate left ventricle myocardium. Samples were snap frozen in liquid nitrogen and stored at − 80 °C or fixed with PFA for histological studies.

### Histology

Left ventricle myocardial tissues were fixed in buffered paraformaldehyde solution (4%) and embedded in paraffin. Then, 2-μm thick sections were placed on adhesive slides and stained with hematoxylin-eosin. Samples were visualized using a Leica microscope (LEICA ICC50 HD) at 400x magnification using standard procedure. All imaging were performed with group identity blinded whereby at least 10 random images were obtained from each slide. Images were then quantified using imaging software (LAS EZ 2.0). The widths of randomly selected cardiomyocytes were measured from 100 LV cardiomyocytes to represent each sample.

Histologic examination of the LV muscles was reviewed by a single expert cardiac pathologist (RW) who was blinded to all other features of the samples characteristics. All samples were evaluated for the presence or absence of the following myocardial features: cardiomyocyte hypertrophy, myofiber disarray, and focal fibrosis. Hypertrophy was diagnosed if myocytes consistently had enlarged and hyperchromatic nuclei, and cell diameters greater than the diameters of 3 red blood cells/RBCs [24]. Myofiber disarray included cellular interlacing, whirling, or herringbone patterns [25]. Myocardium was evaluated for the presence or absence of focal fibrosis. In non-dilated hearts, myocyte diameter is directly proportional to the extent of hypertrophy. Therefore, hypertrophy was considered mild if myocyte overall diameters were 3–4 RBCs, moderate if they were 4–5 RBCs, and severe if they were > 5 RBCs. Myofiber disarray was graded as mild if its extent was 1 to 25% of the myocardial area on the microscopic slide, moderate if 26 to 50%, and severe if > 50%. Focal fibrosis was determined to be absent if there was no focal fibrosis, mild if there was 0 to 5, moderate if there was 6 to 10, and severe if there was > 10 focal fibrosis of the myocardial area. This histology characterization was adaptated from another study with some modification [24].

### RNA extractions and semi-quantitative PCR

RNA from left ventricle myocardial tissue were extracted using TRIsure reagent (Bioline, United Kingdom). Quantification of RNA extracted from the tissue were done using Multimode Microplate Reader at 268/280 nm absorbance spectrophotometry (M200 Pro, Tecan, Morrisville, NC). One Step RT PCR Kit (Bioline, United Kingdom) were used to conduct semiquantitative PCR, GAPDH were used as housekeeping gene. Electrophoresis Gels were visualized using BluePad Detection system and Image J were used for visualization and quantification of PCR band. Table 1 provided lists of primers sequences used in this study.

**Table 1:** Primers used for Semi quantitative-PCR analysis

Gene Symbol	Primer Sequence (5′ to 3′) Upper strand: sense Lower strand: antisense	Product Size (bp)	Annealing (°C)	Cycle	References
αMHC	GAGCAGGAGCTGATCGAGAC	151	60	35	[26]
	CCTCTGCGTTCCTACACTCC				
PIK3CA	ACCTCAGGCTTGAAGAGTGTCG	137	59	35	[27]
	CCGTAAGTCGTCGCCATTTTTA				
mTOR	CTGATGTCATTTATTGGCACAAA	170	57	35	[28]
	CAGGGACTCAGAACACAAATGC				
LC3	GGTCCAGTTGTGCCTTTATTGA	153	59,5	35	[28]
	GTGTGTGGGTTGTGTACGTCG				
p62	CTAGGCATCGAGGTTGACATT	116	56	35	[29]
	CTTGGCTGAGTACCACTCTTATC				
GAPDH	GTTACCAGGGCTGCCTTCTC	177	61	35	[30]
	GATGGTGATGGGTTTCCCGT				

### Statistics

The results of all individual values (*n* = 5) were presented in Additional file 2. SPSS 20.0 software was used for statistical analysis, the results were presented in Additional file 3. The presented results were mean ± standard error of mean (mean ± SEM). One Way ANOVA/Kruskal Wallis were used to examine mean differences between groups with LSD post hoc test (for data with normal distribution) or Mann Whitney test (for data without normal distribution), with 95% confidence interval (*p* < 0.05).

## Results

### Effects of training on heart weight and heart weight/body weight ratio

**Fig. 1:**
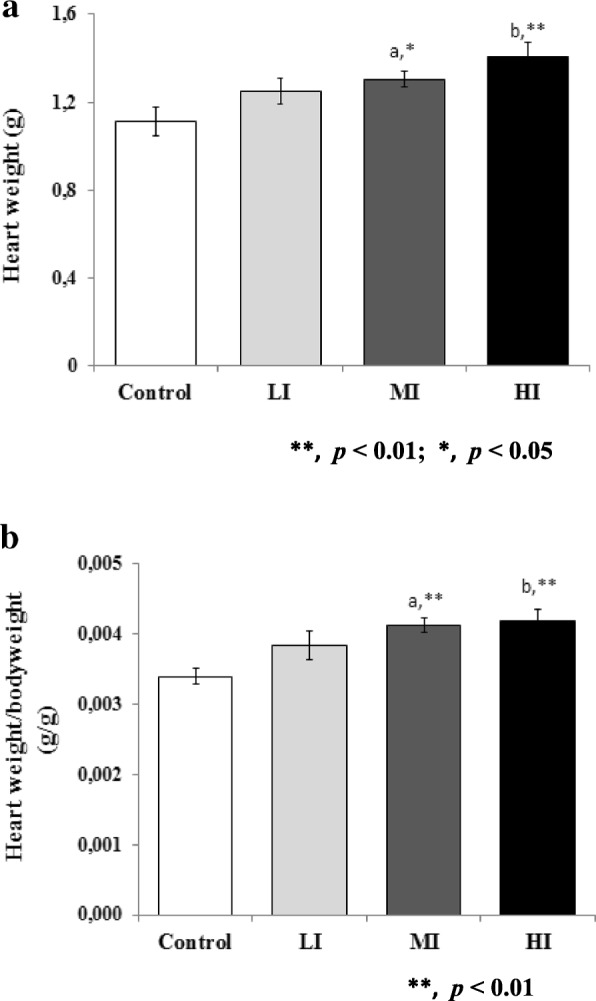
Evaluation of Cardiac Hypertrophy After 8 Weeks of Treadmill Training with Different Intensity. **a** A significant increased of heart weight values were found in MI (**a**) and HI (**b**) compared to control. **b** Heart weight/body weight ratio showed even more significant increase in MI (**a**) and HI (**b**) compared to control. Data was presented as average mean ± standard error of mean (SEM) with *p* < 0.05 considered as significant (*) and *p* < 0.01 considered as very significant (**)

The rats of the four experimental groups had the similar initial body weights (200 ± 50 g). The number of animals in each group included in each analysis is 5/5. Heart weights were recorded after termination and then compared to body weights to examine the ratio. We found a significant difference of heart weight in MI (1.304 ± 0.04) and HI (1.404 ± 0.07), compared to control (1.112 ± 0.07), as showed in Fig. 1a. The heart weight to body weight ratio showed an even more significant increase in MI (0.0041 ± 0.00011) and HI (0.0042 ± 0.00016) compared to control (0.0034 ± 0.00012) (Fig. 1b), reflecting characteristics of cardiac hypertrophy.

### Effects of training on histology

**Fig. 2:**
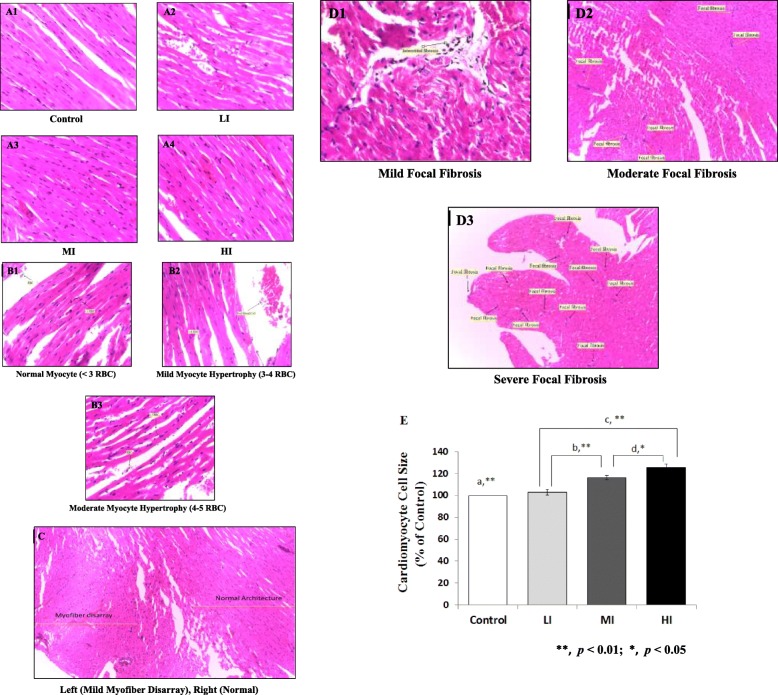
Evaluation of Microscopic Cardiac Hypertrophy After 8 Weeks of Treadmill Training with Different Intensity. [A1–4] Representative photomicrographs of Left Ventricular (LV) myocardium after Hematoxylin and Eosin. Longitudinally oriented cardiomyocytes are displayed for control and exercised rats. [B1–3] Representative photomicrographs of cardiomyocyte hypertrophy found in LI (20%), MI (100%) and HI (100%). Cardiomyocyte cells were compared to RBC (Red blood cell). [C] Representative photomicrograph of mild myofiber disarray in MI (60%) and HI (100%). [D1–3] Representative photomicrographs of focal fibrosis in HI (100%). [E] A significant increased of cardiomyocyte cells size were observed in MI and HI training (**a**) compared to control (100%) and between LI and MI (**b**), LI and HI (**c**), MI and HI (**d**). Data was presented as average mean ± standard error of mean (SEM) with *p* < 0.05 considered as significant (*) and *p* < 0.01 considered as very significant (**)

Training with different intensity resulted in a different histology characteristics presented in Fig. 2a-d and Table 2. For myocyte hypertrophy, we found none in control, 20% mild myocyte hypertrophy in LI, 40% mild and 60% moderate myocyte hypertrophy in MI, and 20% mild and 80% moderate myocyte hypertrophy in HI. As for myofiber disarray, we found none in Control and LI, 60% mild myofiber disarray in MI, and 100% mild myofiber disarray in HI. And for focal fibrosis, we found none in Control, LI, and MI, but 20% mild, 50% moderate, and 20% severe focal fibrosis in HI. We also found a significant increase in percentage cardiomyocyte cell size in MI (116.4 ± 2.04) and HI (125.6 ± 3.03) training compared to control (100%) and LI (103.1 ± 2.38) (Fig. 2e).

**Table 2:** Histologic Characterization of Cardiac Hypertrophy by Different Intensity

Control				
	None	Mild	Moderate	Severe
Myocyte hypertrophy	5 (100%)	0	0	0
Myofiber disarray	5 (100%)	0	0	0
Focal fibrosis	5 (100%)	0	0	0
Low-Intensity				
	None	Mild	Moderate	Severe
Myocyte hypertrophy	4 (80%)	1 (20%)	0	0
Myofiber disarray	5 (100%)	0	0	0
Focal fibrosis	5 (100%)	0	0	0
Moderate-Intensity				
	None	Mild	Moderate	Severe
Myocyte hypertrophy	0	2 (40%)	3 (60%)	0
Myofiber disarray	2 (40%)	3 (60%)	0	0
Focal fibrosis	5 (100%)	0	0	0
High-Intensity				
	None	Mild	Moderate	Severe
Myocyte hypertrophy	0	1 (20%)	4 (80%)	0
Myofiber disarray	0	5 (100%)	0	0
Focal fibrosis	0	1 (20%)	3 (60%)	1 (20%)

### αMHC mRNA expressions in left ventricle myocardium of Wistar rats

**Fig. 3:**
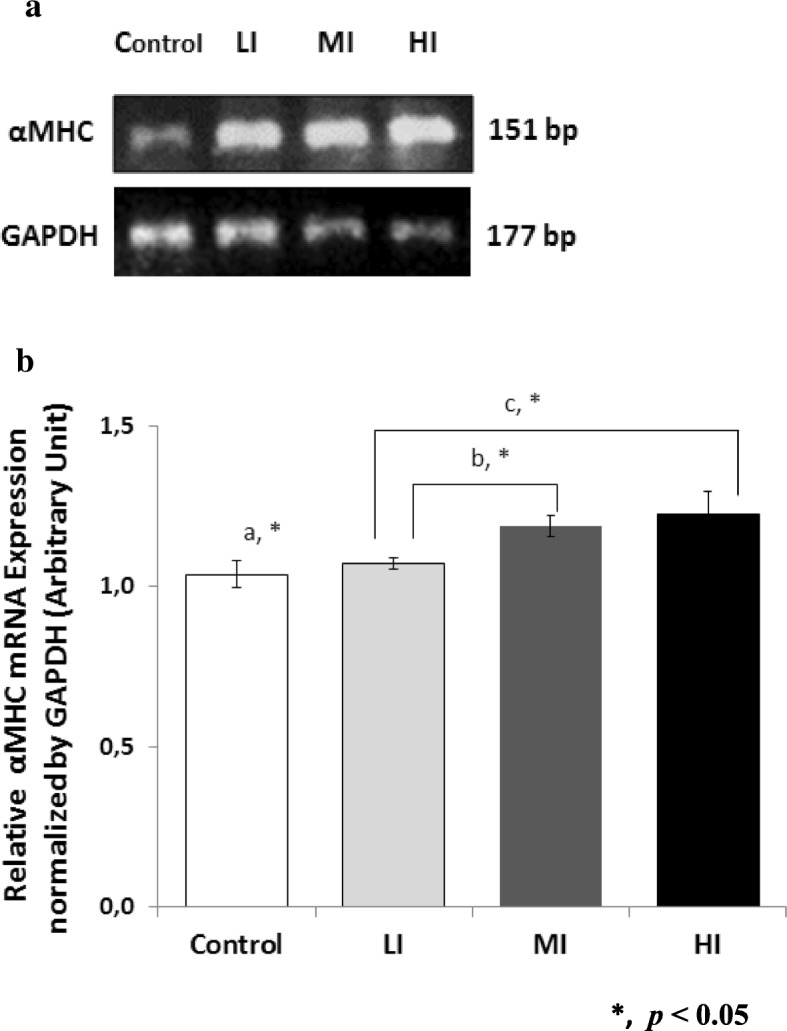
αMHC mRNA Expression in Cardiac Muscles After 8 Weeks of Treadmill Training with Different Intensity. **a** 8 weeks of Treadmill Training with Different Intensity Stimulates αMHC mRNA Expression in Rat Cardiac Muscle (Left ventricle myocardium). **b** A significant increase of αMHC mRNA Expression were found in MI compared to control (a), between LI and MI (b), and between LI and HI (c). αMHC mRNA expression in HI is increased with p = 0.076, compared to control. Data was presented as average mean ± standard error of mean (SEM) with *p* < 0.05 considered as significant (*)

In order to confirm cardiac hypertrophy in training heart, we also examined αMHC gene expression using semi-quantitative PCR. PCR bands of αMHC were normalized using GAPDH. The result is presented in Fig. 3. In MI and HI groups, αMHC gene expression were increased (MI 1.1 fold, *p <* 0.05 and HI 1.2 fold, *p* = 0.076) compared to control.

### PIK3CA and mTOR mRNA expressions in left ventricle myocardium of wistar rats

**Fig. 4:**
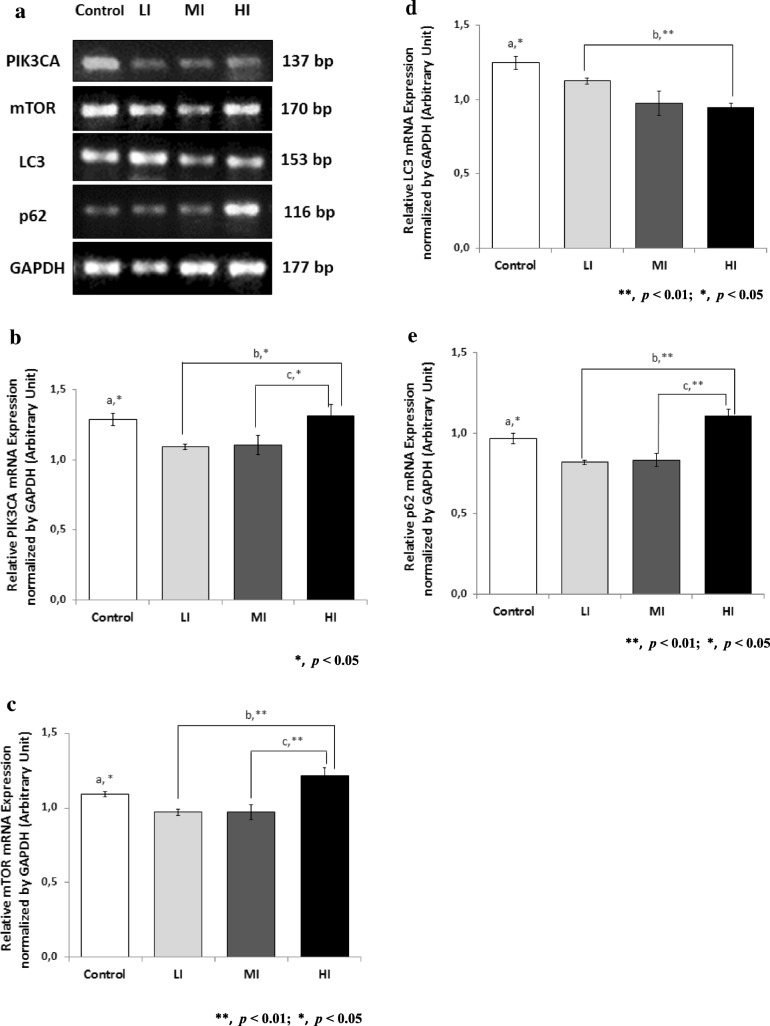
Autophagy Related mRNA Expression in Cardiac Muscles After 8 Weeks of Treadmill Training with Different Intensity. **a** 8 weeks of Treadmill Training with Different Intensity Modulates PIK3CA, mTOR, LC3, p62 mRNA Expression in Rat Cardiac Muscle (Left ventricle myocardium). **b** A significant decrease of PIK3CA mRNA Expression were found in LI and MI compared to control (a), and between LI and HI (b), MI and HI (c). **c** mTOR mRNA expression also significantly decreased in LI, MI and increased in HI compared to control (a), between LI and HI (b) and MI and HI (c). **d** LC3 mRNA expression also significantly decreased in LI, MI and HI compared to control (a), and between LI and HI (b). **e** p62 mRNA expression also significantly decreased in LI, MI and increased in HI compared to control (a), between LI and HI (b) and MI and HI (c). Data was presented as average mean ± standard error of mean (SEM) with *p* < 0.05 considered as significant (*), and *p* < 0.01 considered as very significant (**)

We also examined PIK3CA and mTOR gene expressions in left ventricle myocardium of Wistar rats using semi-quantitative PCR. PCR bands of PIK3A and mTOR were normalized using GAPDH. The result is presented in Fig. 4a-c. Training with low and moderate intensities significantly decreased PIK3CA gene expression (LI 0.8, MI 0.9, *p* < 0.05). mTOR gene expression also significantly decreased (LI 0.9 fold, MI 0.9 fold *p <* 0.05) compared to control, but interestingly, training with high intensity increased mTOR gene expression (HI 1.1, *p* < 0.05).

### LC3 and p62 mRNA expressions in left ventricle myocardium of wistar rats

We examined the autophagy related gene expression using semi-quantitative PCR. PCR bands of LC3 and p62 were normalized using GAPDH. The result is presented in Fig. 4a, d-e. Training significantly decreased expression of autophagy gene LC3 (LI 0.9 fold, MI 0.8 fold, HI 0.8 fold, *p <* 0.05) in left ventricle myocardium compared to control. On the other side, training significantly decreased p62 gene expression (LI 0.8 fold, MI 0.9 fold, *p <* 0.05), and increased p62 gene expression (HI 1.1 fold, *p <* 0.05).

## Discussion

The heart contains multiple cell types, including myocytes (muscle cells), nonmyocytes (fibroblasts, endothelial cells, mast cells, leukocytes, vascular smooth muscle cells/mural cells), and the surrounding extracellular matrix. It is well accepted that cardiac myocytes represent 30–40% of the cell population in the adult rodent and human heart, so it represents 70–80% of the heart’s volume. Hypertrophy of cardiac myocytes appears to be the predominant factor that contributes to heart enlargement as a response to chronic training [9, 31].

Different intensity of training may create different response of cardiac hypertrophy. In response to moderate training, cardiac hypertrophy occurs as an adaptive physiological response that is associated with normal or improved cardiac function [32, 33]. However, it is unavoided that endurance training have correlation with maladaptive responses in cardiac hypertrophy which is involving abnormal dynamic regulation of blood pressure, and histological rearrangement forming fibrotic tissue. Benito et al. had reported that fibrotic tissue in myocardial parenchyma as a consequence of regenerative and adaptive process can cause electrical impulse reentry lead to arrhythmogenicity [34].

In this present study, we found cardiac hypertrophy in MI and HI, supported by increased heart weight, heart weight/body weight ratio, histology, % cardiomyocyte cell size and increased αMHC gene expression. Treadmill is one of animal modes that can induce physiological cardiac hypertrophy, measured both at the whole heart, ventricle, and individual cardiomyocyte level. This hypertrophy can be detected after 4 weeks of training, and reaches plateau after a few months if the training is sustained [35]. Our study also finds focal fibrosis in HI group, which may be associated with maladaptive training-induced cardiac remodeling. At least one animal study suggests that long-term, intensive endurance training (treadmill running for 16 weeks) is shown to be associated with fibrosis within the right ventricle. However in this study, the most marker of myocardial fibrosis returned to baseline after discontinuation of training. This suggests that the fibrosis is of the reactive phenotype, which is reversible after detraining [33, 34].

Our study also shows an altered autophagy gene expression based on different intensity. LI and MI may increase autophagy gene expression, supported by decreased LC3, p62, mTOR, and PIK3CA relative ratio normalized by GAPDH. However, HI may decrease autophagy, supported by decreased LC3 and increased p62, mTOR relative ratio normalized by GAPDH. Recent studies have shown that endurance training can enhance autophagy in cardiac muscles [16, 36]. Increased autophagy in LI and decreased autophagy in HI may be associated with a decrease and increase of protein synthesis that correlates with cardiac hypertrophy.

The best signaling cascades responsible for mediating physiological cardiac hypertrophy is IGF1-PI3KCA/PI3K(p110α)-Akt pathway. mTOR is a downstream pathway of IGF1-PIK3CA that has long been considered to be a potent autophagy regulator, as inhibition or activation of mTOR regulates autophagy [37]. In this study, we found a decrease of mTOR and PIK3CA gene expression in LI and MI, suggested autophagy may be increased in cardiac muscle as a result of training-induced adaptation. Interestingly, we also found cardiac hypertrophy in MI training, this suggest that cardiac hypertrophy may occur from other pathway, like gp130/JAK/STAT pathway, but a future study is needed to confirm this hypothesis.

The limitation of this study is that we did not examine cardiac function and collagen deposition in left ventricle. There are also possibilities that the physiological cardiac hypertrophy after training could be affected by other factors, such as hormonal [38], genetic [39], or other metabolic factors [40]. This study has been demonstrated the effect of different intensity of treadmill training on cardiac hypertrophy of male wistar rats, and may not be applicable in humans. Further study has to be carried out to know the effect of different treadmill intensity on cardiac hypertrophy in humans, in order to determine how much training is optimal for cardiovascular benefit, which is correlated with cardiac physiological adaptation after training with autophagy regulation involvement [8, 9].

## Conclusion

In summary, different training intensity might stimulate different process of cardiac hypertrophy that correlates with autophagy. LI training may increase autophagy related gene expression, but not inducing cardiac hypertrophy. MI training may induce cardiac hypertrophy with increased autophagy related gene expression, while HI training may induce cardiac hypertrophy with decreased autophagy related gene expression.

## Additional files

Additional file 1: **Figure S1.** Experimental Design of the Research. Animals were randomly allocated to 4 groups upon arrival. Three treadmill training intensities (Low-Intensity/LI, Moderate-Intensity/MI, and High-Intensity/HI) and one group without treadmill training/Control, were compared. (DOCX 44 kb)

Additional file 2: Individual Data of The Research. (DOCX 27 kb)

Additional file 3: Statistical Results of The Research. (DOCX 104 kb)

## Data Availability

The datasets used and/or analysed during the current study are available from the corresponding author on reasonable request.

## Abbreviations

HI: High Intensity
LI: Low Intensity
MI: Moderate Intensity
