# Supplementary material for: Cardiac hypertrophy is stimulated by altered training intensity and correlates with autophagy modulation in male Wistar rats
Source: BMC Sports Sci Med Rehabil. 2019 Jun 10;11:9. doi: 10.1186/s13102-019-0121-0 (PMC6558762; doi:10.1186/s13102-019-0121-0)
Supplement: Supplementary file 1 — Figure S1. Experimental Design of the Research. Animals were randomly allocated to 4 groups upon arrival. Three treadmill training intensities (Low-Intensity/LI, Moderate-Intensity/MI, and High-Intensity/HI) and one group without treadmill training/Control, were compared. (DOCX 44 kb) [file 13102_2019_121_MOESM1_ESM.docx]

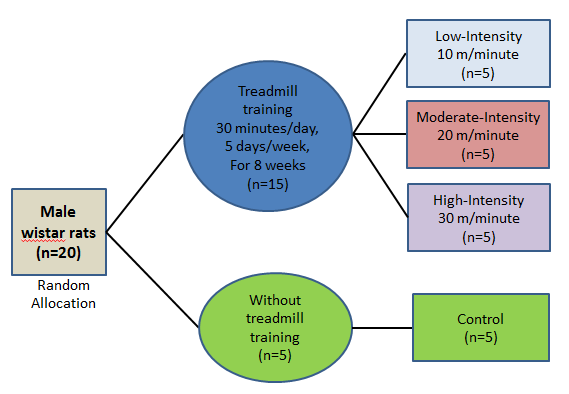


**Additional file 1: Figure S1**

**Experimental Design of the Research. Animals were randomly allocated to 4 groups upon arrival. Three treadmill training intensities (Low-Intensity/LI, Moderate-Intensity/MI, and High-Intensity/HI) and one group without treadmill training/Control, were compared.**
