# Supplementary material for: Cardiac hypertrophy is stimulated by altered training intensity and correlates with autophagy modulation in male Wistar rats
Source: BMC Sports Sci Med Rehabil. 2019 Jun 10;11:9. doi: 10.1186/s13102-019-0121-0 (PMC6558762; doi:10.1186/s13102-019-0121-0)
Supplement: Supplementary file 2 — Individual Data of The Research. (DOCX 27 kb) [file 13102_2019_121_MOESM2_ESM.docx]

Heart weight of male wistar rats after Treadmill Training with Different Intensity

| Heart weight (gram) | Control (gram) | LI (gram) | MI (gram) | HI (gram) |
| --- | --- | --- | --- | --- |
| 1 | 1.12 | 1.37 | 1.33 | 1.54 |
| 2 | 1.21 | 1.37 | 1.30 | 1.59 |
| 3 | 1.29 | 1.27 | 1.35 | 1.29 |
| 4 | 0.93 | 1.09 | 1.37 | 1.25 |
| 5 | 1.01 | 1.15 | 1.17 | 1.35 |
| Mean | 1.112 | 1.25 | 1.304 | 1.404 |
| SEM | 0.07 | 0.06 | 0.04 | 0.07 |

Heart weight/weights Ratio of male wistar rats after Treadmill Training with Different Intensity

| Heart weight/weight Ratio (g/g) | Control (g/g) | LI (g/g) | MI (g/g) | HI (g/g) |
| --- | --- | --- | --- | --- |
| 1 | 0.00314 | 0.00322 | 0.00420 | 0.00467 |
| 2 | 0.00360 | 0.00434 | 0.00425 | 0.00440 |
| 3 | 0.00348 | 0.00385 | 0.00408 | 0.00398 |
| 4 | 0.00368 | 0.00426 | 0.00438 | 0.00377 |
| 5 | 0.00311 | 0.00356 | 0.00375 | 0.00412 |
| Mean | 0.00340 | 0.00384 | 0.00413 | 0.00419 |
| SEM | 0.00012 | 0.00021 | 0.00011 | 0.00016 |

Cardiomyocyte Cell Size (% of Control) of Male Wistar rats after Treadmill Training with Different Intensity

| Cardiomyocyte Cell Size (% of Control) | Control (%) | LI (%)] | MI (%) | HI (%) |
| --- | --- | --- | --- | --- |
| 1 | 100 | 98.0 | 111.4 | 114.4 |
| 2 | 100 | 106.5 | 122.5 | 127.3 |
| 3 | 100 | 107.7 | 113.0 | 132.5 |
| 4 | 100 | 96.6 | 115.6 | 125.2 |
| 5 | 100 | 106.5 | 119.4 | 128.4 |
| Mean | 100 | 103.1 | 116.4 | 125.6 |
| SEM | 0.00 | 2.38 | 2.04 | 3.03 |

αMHC mRNA Expression normalized by GAPDH in left myocardial tissue of male wistar rats after Treadmill Training with Different Intensity

| Relative mRNA Expression | Control | LI | MI | HI |
| --- | --- | --- | --- | --- |
| 1 | 0.943 | 1.093 | 1.165 | 1.091 |
| 2 | 1.142 | 1.042 | 1.123 | 1.456 |
| 3 | 1.132 | 1.036 | 1.136 | 1.166 |
| 4 | 1.012 | 1.131 | 1.298 | 1.326 |
| 5 | 0.966 | 1.058 | 1.237 | 1.106 |
| Mean | 1.039 | 1.072 | 1.192 | 1.229 |
| SEM | 0.04 | 0.02 | 0.03 | 0.07 |

PIK3CA mRNA Expression normalized by GAPDH in left myocardial tissue of male wistar rats Treadmill Training with Different Intensity

| Relative mRNA Expression | | | Control | | LI | | MI | HI |
| --- | --- | --- | --- | --- | --- | --- | --- | --- |
| 1 | | | 1.329 | | 1.071 | | 1.171 | 1.117 |
| 2 | | | 1.340 | | 1.072 | | 0.872 | 1.220 |
| 3 | | | 1.117 | | 1.114 | | 1.063 | 1.435 |
| 4 | | | 1.371 | | 1.163 | | 1.271 | 1.559 |
| 5 | | | 1.282 | | 1.046 | | 1.148 | 1.239 |
| Mean | | | 1.288 | | 1.093 | | 1.105 | 1.314 |
| SEM | | | 0.05 | | 0.02 | | 0.07 | 0.08 |
|  |  |  | |  | |  |  |  |

mTOR mRNA Expression normalized by GAPDH in left myocardial tissue of male wistar rats Treadmill Training with Different Intensity

| Relative mRNA Expression | | Control | | LI | | MI | | HI |
| --- | --- | --- | --- | --- | --- | --- | --- | --- |
| 1 | | 1.126 | | 0.961 | | 1.031 | | 1.096 |
| 2 | | 1.102 | | 1.046 | | 0.807 | | 1.395 |
| 3 | | 1.122 | | 0.923 | | 0.904 | | 1.151 |
| 4 | | 1.054 | | 0.986 | | 1.045 | | 1.251 |
| 5 | | 1.050 | | 0.944 | | 1.066 | | 1.195 |
| Mean | | 1.091 | | 0.972 | | 0.971 | | 1.218 |
| SEM | | 0.02 | | 0.02 | | 0.05 | | 0.05 |
|  |  | |  | |  | |  |  |

LC3 mRNA Expression normalized by GAPDH in left myocardial tissue of male wistar rats Treadmill Training with Different Intensity

| Relative mRNA Expression | Control | LI | MI | HI |
| --- | --- | --- | --- | --- |
| 1 | 1.238 | 1.121 | 1.095 | 0.869 |
| 2 | 1.262 | 1.096 | 0.792 | 0.905 |
| 3 | 1.396 | 1.210 | 0.759 | 0.949 |
| 4 | 1.232 | 1.118 | 1.112 | 0.978 |
| 5 | 1.120 | 1.079 | 1.124 | 1.031 |
| Mean | 1.250 | 1.125 | 0.976 | 0.946 |
| SEM | 0.04 | 0.02 | 0.08 | 0.03 |

p62 mRNA Expression normalized by GAPDH in left myocardial tissue of male wistar rats Treadmill Training with Different Intensity

| Relative mRNA Expression | Control | LI | MI | HI |
| --- | --- | --- | --- | --- |
| 1 | 0.849 | 0.799 | 0.977 | 1.059 |
| 2 | 0.997 | 0.864 | 0.741 | 1.207 |
| 3 | 0.933 | 0.790 | 0.758 | 0.971 |
| 4 | 1.012 | 0.837 | 0.822 | 1.128 |
| 5 | 1.043 | 0.803 | 0.870 | 1.171 |
| Mean | 0.967 | 0.819 | 0.834 | 1.107 |
| SEM | 0.03 | 0.01 | 0.04 | 0.04 |
