# Supplementary material for: Cardiac hypertrophy is stimulated by altered training intensity and correlates with autophagy modulation in male Wistar rats
Source: BMC Sports Sci Med Rehabil. 2019 Jun 10;11:9. doi: 10.1186/s13102-019-0121-0 (PMC6558762; doi:10.1186/s13102-019-0121-0)
Supplement: Supplementary file 3 — Statistical Results of The Research. (DOCX 104 kb) [file 13102_2019_121_MOESM3_ESM.docx]

Statistical Results

1. Heart Weight

**Oneway**

| **Descriptives** | | | | | | | | | | | | |
| --- | --- | --- | --- | --- | --- | --- | --- | --- | --- | --- | --- | --- |
| Heartweight | | | | | | | | | | | | |
|  | N | Mean | | Std. Deviation | | | Std. Error | | 95% Confidence Interval for Mean | | Minimum | Maximum |
|  |  |  |  |  |  |  |  |  | Lower Bound | Upper Bound |  |  |
| 1 | 5 | 1,11200 | | ,145671 | | | ,065146 | | ,93113 | 1,29287 | ,930 | 1,290 |
| 2 | 5 | 1,25000 | | ,127279 | | | ,056921 | | 1,09196 | 1,40804 | 1,090 | 1,370 |
| 3 | 5 | 1,30400 | | ,079246 | | | ,035440 | | 1,20560 | 1,40240 | 1,170 | 1,370 |
| 4 | 5 | 1,40400 | | ,152250 | | | ,068088 | | 1,21496 | 1,59304 | 1,250 | 1,590 |
| Total | 20 | 1,26750 | | ,160554 | | | ,035901 | | 1,19236 | 1,34264 | ,930 | 1,590 |
| **Test of Homogeneity of Variances** | | | | | | | |  |  |  |  |  |
| Heartweight | | | | | | | |  |  |  |  |  |
| Levene Statistic | | | df1 | | df2 | Sig. | |  |  |  |  |  |
| 1,596 | | | 3 | | 16 | ,230 | |  |  |  |  |  |

| **Tests of Normality** | | | | | | | |
| --- | --- | --- | --- | --- | --- | --- | --- |
|  | Kelompok | Kolmogorov-Smirnov^a^ | | | Shapiro-Wilk | | |
|  |  | Statistic | df | Sig. | Statistic | df | Sig. |
| Heartweight | 1 | ,158 | 5 | ,200^*^ | ,974 | 5 | ,901 |
|  | 2 | ,227 | 5 | ,200^*^ | ,883 | 5 | ,323 |
|  | 3 | ,280 | 5 | ,200^*^ | ,837 | 5 | ,157 |
|  | 4 | ,239 | 5 | ,200^*^ | ,884 | 5 | ,329 |
| a. Lilliefors Significance Correction  *. This is a lower bound of the true significance.   \| **ANOVA** \| \| \| \| \| \| \| --- \| --- \| --- \| --- \| --- \| --- \| \| Heartweight \| \| \| \| \| \| \|  \| Sum of Squares \| df \| Mean Square \| F \| Sig. \| \| Between Groups \| ,222 \| 3 \| ,074 \| 4,431 \| ,019 \| \| Within Groups \| ,268 \| 16 \| ,017 \|  \|  \| \| Total \| ,490 \| 19 \|  \|  \|  \| | | | | | | | |

**Post Hoc Tests**

| **Multiple Comparisons** | | | | | | |
| --- | --- | --- | --- | --- | --- | --- |
| Heartweight  LSD | | | | | | |
| (I) Kelompok | (J) Kelompok | Mean Difference (I-J) | Std. Error | Sig. | 95% Confidence Interval | |
|  |  |  |  |  | Lower Bound | Upper Bound |
| 1 | 2 | -,138000 | ,081780 | ,111 | -,31137 | ,03537 |
|  | 3 | -,192000^*^ | ,081780 | ,032 | -,36537 | -,01863 |
|  | 4 | -,292000^*^ | ,081780 | ,003 | -,46537 | -,11863 |
| 2 | 1 | ,138000 | ,081780 | ,111 | -,03537 | ,31137 |
|  | 3 | -,054000 | ,081780 | ,518 | -,22737 | ,11937 |
|  | 4 | -,154000 | ,081780 | ,078 | -,32737 | ,01937 |
| 3 | 1 | ,192000^*^ | ,081780 | ,032 | ,01863 | ,36537 |
|  | 2 | ,054000 | ,081780 | ,518 | -,11937 | ,22737 |
|  | 4 | -,100000 | ,081780 | ,239 | -,27337 | ,07337 |
| 4 | 1 | ,292000^*^ | ,081780 | ,003 | ,11863 | ,46537 |
|  | 2 | ,154000 | ,081780 | ,078 | -,01937 | ,32737 |
|  | 3 | ,100000 | ,081780 | ,239 | -,07337 | ,27337 |
| *. The mean difference is significant at the 0.05 level. | | | | | | |

1. HW/Weight

**Oneway**

| **Descriptives** | | | | | | | | |
| --- | --- | --- | --- | --- | --- | --- | --- | --- |
| Heartweightweight | | | | | | | | |
|  | N | Mean | Std. Deviation | Std. Error | 95% Confidence Interval for Mean | | Minimum | Maximum |
|  |  |  |  |  | Lower Bound | Upper Bound |  |  |
| 1 | 5 | ,0033998 | ,00026316 | ,00011769 | ,0030731 | ,0037266 | ,00311 | ,00368 |
| 2 | 5 | ,0038436 | ,00047108 | ,00021067 | ,0032587 | ,0044285 | ,00322 | ,00434 |
| 3 | 5 | ,0041299 | ,00023789 | ,00010639 | ,0038345 | ,0044253 | ,00375 | ,00438 |
| 4 | 5 | ,0041867 | ,00035450 | ,00015854 | ,0037465 | ,0046269 | ,00377 | ,00467 |
| Total | 20 | ,0038900 | ,00044920 | ,00010044 | ,0036798 | ,0041002 | ,00311 | ,00467 |

| **Test of Homogeneity of Variances** | | | |
| --- | --- | --- | --- |
| Heartweightweight | | | |
| Levene Statistic | df1 | df2 | Sig. |
| 1,219 | 3 | 16 | ,335 |

| **Tests of Normality** | | | | | | | |
| --- | --- | --- | --- | --- | --- | --- | --- |
|  | Kelompok | Kolmogorov-Smirnov^a^ | | | Shapiro-Wilk | | |
|  |  | Statistic | df | Sig. | Statistic | df | Sig. |
| Heartweightweight | 1 | ,241 | 5 | ,200^*^ | ,865 | 5 | ,248 |
|  | 2 | ,210 | 5 | ,200^*^ | ,938 | 5 | ,650 |
|  | 3 | ,215 | 5 | ,200^*^ | ,925 | 5 | ,566 |
|  | 4 | ,179 | 5 | ,200^*^ | ,978 | 5 | ,926 |
| a. Lilliefors Significance Correction  *. This is a lower bound of the true significance | | | | | | | |

| **ANOVA** | | | | | |
| --- | --- | --- | --- | --- | --- |
| Heartweightweight | | | | | |
|  | Sum of Squares | df | Mean Square | F | Sig. |
| Between Groups | ,000 | 3 | ,000 | 5,464 | ,009 |
| Within Groups | ,000 | 16 | ,000 |  |  |
| Total | ,000 | 19 |  |  |  |

**Post Hoc Test**

| **Multiple Comparisons** | | | | | | |
| --- | --- | --- | --- | --- | --- | --- |
| Heartweightweight  LSD | | | | | | |
| (I) Kelompok | (J) Kelompok | Mean Difference (I-J) | Std. Error | Sig. | 95% Confidence Interval | |
|  |  |  |  |  | Lower Bound | Upper Bound |
| 1 | 2 | -,00044379 | ,00021758 | ,058 | -,0009051 | ,0000175 |
|  | 3 | -,00073008^*^ | ,00021758 | ,004 | -,0011913 | -,0002688 |
|  | 4 | -,00078688^*^ | ,00021758 | ,002 | -,0012481 | -,0003256 |
| 2 | 1 | ,00044379 | ,00021758 | ,058 | -,0000175 | ,0009051 |
|  | 3 | -,00028628 | ,00021758 | ,207 | -,0007475 | ,0001750 |
|  | 4 | -,00034308 | ,00021758 | ,134 | -,0008043 | ,0001182 |
| 3 | 1 | ,00073008^*^ | ,00021758 | ,004 | ,0002688 | ,0011913 |
|  | 2 | ,00028628 | ,00021758 | ,207 | -,0001750 | ,0007475 |
|  | 4 | -,00005680 | ,00021758 | ,797 | -,0005181 | ,0004045 |
| 4 | 1 | ,00078688^*^ | ,00021758 | ,002 | ,0003256 | ,0012481 |
|  | 2 | ,00034308 | ,00021758 | ,134 | -,0001182 | ,0008043 |
|  | 3 | ,00005680 | ,00021758 | ,797 | -,0004045 | ,0005181 |
| *. The mean difference is significant at the 0.05 level. | | | | | | |

1. Cardiomyocytes Cell Size

| **Descriptives** | | | | | | | | |
| --- | --- | --- | --- | --- | --- | --- | --- | --- |
| CardiomycoyteCellSize | | | | | | | | |
|  | N | Mean | Std. Deviation | Std. Error | 95% Confidence Interval for Mean | | Minimum | Maximum |
|  |  |  |  |  | Lower Bound | Upper Bound |  |  |
| 1 | 5 | 100,0000 | ,00000 | ,00000 | 100,0000 | 100,0000 | 100,00 | 100,00 |
| 2 | 5 | 103,0563 | 5,31425 | 2,37661 | 96,4578 | 109,6548 | 96,55 | 107,68 |
| 3 | 5 | 116,3882 | 4,55343 | 2,03636 | 110,7344 | 122,0421 | 111,44 | 122,51 |
| 4 | 5 | 125,5588 | 6,78602 | 3,03480 | 117,1328 | 133,9847 | 114,38 | 132,50 |
| Total | 20 | 111,2508 | 11,48066 | 2,56715 | 105,8777 | 116,6239 | 96,55 | 132,50 |

| **Test of Homogeneity of Variances** | | | |
| --- | --- | --- | --- |
| CardiomycoyteCellSize | | | |
| Levene Statistic | df1 | df2 | Sig. |
| 3,809 | 3 | 16 | ,031 |

**Kruskal-Wallis Test**

| **Ranks** | | | |
| --- | --- | --- | --- |
|  | Kelompok | N | Mean Rank |
| CardiomycoyteCellSize | 1 | 5 | 5,00 |
|  | 2 | 5 | 6,00 |
|  | 3 | 5 | 13,60 |
|  | 4 | 5 | 17,40 |
|  | Total | 20 |  |

| **Test Statistics^a,b^** | |
| --- | --- |
|  | CardiomycoyteCellSize |
| Chi-Square | 15,624 |
| df | 3 |
| Asymp. Sig. | ,001 |
| a. Kruskal Wallis Test  b. Grouping Variable: Kelompok | |

**Mann-Whitney Test**

| **Ranks** | | | | |
| --- | --- | --- | --- | --- |
|  | Kelompok | N | Mean Rank | Sum of Ranks |
| CardiomycoyteCellSize | 1 | 5 | 5,00 | 25,00 |
|  | 2 | 5 | 6,00 | 30,00 |
|  | Total | 10 |  |  |

| **Test Statistics^b^** | |
| --- | --- |
|  | CardiomycoyteCellSize |
| Mann-Whitney U | 10,000 |
| Wilcoxon W | 25,000 |
| Z | -,557 |
| Asymp. Sig. (2-tailed) | ,577 |
| Exact Sig. [2*(1-tailed Sig.)] | ,690^a^ |
| a. Not corrected for ties.  b. Grouping Variable: Kelompok | |

| **Ranks** | | | | |
| --- | --- | --- | --- | --- |
|  | Kelompok | N | Mean Rank | Sum of Ranks |
| CardiomycoyteCellSize | 1 | 5 | 3,00 | 15,00 |
|  | 3 | 5 | 8,00 | 40,00 |
|  | Total | 10 |  |  |

| **Test Statistics^b^** | |
| --- | --- |
|  | CardiomycoyteCellSize |
| Mann-Whitney U | ,000 |
| Wilcoxon W | 15,000 |
| Z | -2,785 |
| Asymp. Sig. (2-tailed) | ,005 |
| Exact Sig. [2*(1-tailed Sig.)] | ,008^a^ |
| a. Not corrected for ties.  b. Grouping Variable: Kelompok | |

| **Ranks** | | | | |
| --- | --- | --- | --- | --- |
|  | Kelompok | N | Mean Rank | Sum of Ranks |
| CardiomycoyteCellSize | 1 | 5 | 3,00 | 15,00 |
|  | 4 | 5 | 8,00 | 40,00 |
|  | Total | 10 |  |  |

| **Test Statistics^b^** | |
| --- | --- |
|  | CardiomycoyteCellSize |
| Mann-Whitney U | ,000 |
| Wilcoxon W | 15,000 |
| Z | -2,785 |
| Asymp. Sig. (2-tailed) | ,005 |
| Exact Sig. [2*(1-tailed Sig.)] | ,008^a^ |
| a. Not corrected for ties.  b. Grouping Variable: Kelompok | |

| **Ranks** | | | | |
| --- | --- | --- | --- | --- |
|  | Kelompok | N | Mean Rank | Sum of Ranks |
| CardiomycoyteCellSize | 2 | 5 | 3,00 | 15,00 |
|  | 3 | 5 | 8,00 | 40,00 |
|  | Total | 10 |  |  |

| **Test Statistics^b^** | |
| --- | --- |
|  | CardiomycoyteCellSize |
| Mann-Whitney U | ,000 |
| Wilcoxon W | 15,000 |
| Z | -2,611 |
| Asymp. Sig. (2-tailed) | ,009 |
| Exact Sig. [2*(1-tailed Sig.)] | ,008^a^ |
| a. Not corrected for ties.  b. Grouping Variable: Kelompok | |

| **Ranks** | | | | |
| --- | --- | --- | --- | --- |
|  | Kelompok | N | Mean Rank | Sum of Ranks |
| CardiomycoyteCellSize | 2 | 5 | 3,00 | 15,00 |
|  | 4 | 5 | 8,00 | 40,00 |
|  | Total | 10 |  |  |

| **Test Statistics^b^** | |
| --- | --- |
|  | CardiomycoyteCellSize |
| Mann-Whitney U | ,000 |
| Wilcoxon W | 15,000 |
| Z | -2,611 |
| Asymp. Sig. (2-tailed) | ,009 |
| Exact Sig. [2*(1-tailed Sig.)] | ,008^a^ |
| a. Not corrected for ties.  b. Grouping Variable: Kelompok | |

| **Ranks** | | | | |
| --- | --- | --- | --- | --- |
|  | Kelompok | N | Mean Rank | Sum of Ranks |
| CardiomycoyteCellSize | 3 | 5 | 3,60 | 18,00 |
|  | 4 | 5 | 7,40 | 37,00 |
|  | Total | 10 |  |  |

| **Test Statistics^b^** | |
| --- | --- |
|  | CardiomycoyteCellSize |
| Mann-Whitney U | 3,000 |
| Wilcoxon W | 18,000 |
| Z | -1,984 |
| Asymp. Sig. (2-tailed) | ,047 |
| Exact Sig. [2*(1-tailed Sig.)] | ,056^a^ |
| a. Not corrected for ties.  b. Grouping Variable: Kelompok | |

1. AlphaMHC

**Oneway**

| **Descriptives** | | | | | | | | |
| --- | --- | --- | --- | --- | --- | --- | --- | --- |
| alphamhc | | | | | | | | |
|  | N | Mean | Std. Deviation | Std. Error | 95% Confidence Interval for Mean | | Minimum | Maximum |
|  |  |  |  |  | Lower Bound | Upper Bound |  |  |
| 1 | 5 | 1,03909 | ,092989 | ,041586 | ,92363 | 1,15455 | ,943 | 1,142 |
| 2 | 5 | 1,07180 | ,039819 | ,017808 | 1,02236 | 1,12124 | 1,036 | 1,131 |
| 3 | 5 | 1,19165 | ,073956 | ,033074 | 1,09982 | 1,28348 | 1,123 | 1,298 |
| 4 | 5 | 1,22897 | ,157343 | ,070366 | 1,03360 | 1,42433 | 1,091 | 1,456 |
| Total | 20 | 1,13288 | ,123098 | ,027525 | 1,07526 | 1,19049 | ,943 | 1,456 |

| **Test of Homogeneity of Variances** | | | |
| --- | --- | --- | --- |
| alphamhc | | | |
| Levene Statistic | df1 | df2 | Sig. |
| 5,693 | 3 | 16 | ,008 |

**Kruskal-Wallis Test**

| **Ranks** | | | |
| --- | --- | --- | --- |
|  | Kelompok | N | Mean Rank |
| alphamhc | 1 | 5 | 6,40 |
|  | 2 | 5 | 6,80 |
|  | 3 | 5 | 14,60 |
|  | 4 | 5 | 14,20 |
|  | Total | 20 |  |

| **Test Statistics^a,b^** | |
| --- | --- |
|  | alphamhc |
| Chi-Square | 8,714 |
| df | 3 |
| Asymp. Sig. | ,033 |
| a. Kruskal Wallis Test  b. Grouping Variable: Kelompok | |

**Mann-Whitney Test**

| **Ranks** | | | | |
| --- | --- | --- | --- | --- |
|  | Kelompok | N | Mean Rank | Sum of Ranks |
| alphamhc | 1 | 5 | 5,00 | 25,00 |
|  | 2 | 5 | 6,00 | 30,00 |
|  | Total | 10 |  |  |

| **Test Statistics^b^** | |
| --- | --- |
|  | alphamhc |
| Mann-Whitney U | 10,000 |
| Wilcoxon W | 25,000 |
| Z | -,522 |
| Asymp. Sig. (2-tailed) | ,602 |
| Exact Sig. [2*(1-tailed Sig.)] | ,690^a^ |
| a. Not corrected for ties.  b. Grouping Variable: Kelompok | |

| **Ranks** | | | | |
| --- | --- | --- | --- | --- |
|  | Kelompok | N | Mean Rank | Sum of Ranks |
| alphamhc | 1 | 5 | 3,60 | 18,00 |
|  | 3 | 5 | 7,40 | 37,00 |
|  | Total | 10 |  |  |

| **Test Statistics^b^** | |
| --- | --- |
|  | alphamhc |
| Mann-Whitney U | 3,000 |
| Wilcoxon W | 18,000 |
| Z | -1,984 |
| Asymp. Sig. (2-tailed) | ,047 |
| Exact Sig. [2*(1-tailed Sig.)] | ,056^a^ |
| a. Not corrected for ties.  b. Grouping Variable: Kelompok | |

| **Ranks** | | | | |
| --- | --- | --- | --- | --- |
|  | Kelompok | N | Mean Rank | Sum of Ranks |
| alphamhc | 1 | 5 | 3,80 | 19,00 |
|  | 4 | 5 | 7,20 | 36,00 |
|  | Total | 10 |  |  |

| **Test Statistics^b^** | |
| --- | --- |
|  | alphamhc |
| Mann-Whitney U | 4,000 |
| Wilcoxon W | 19,000 |
| Z | -1,776 |
| Asymp. Sig. (2-tailed) | ,076 |
| Exact Sig. [2*(1-tailed Sig.)] | ,095^a^ |
| a. Not corrected for ties.  b. Grouping Variable: Kelompok | |

| **Ranks** | | | | |
| --- | --- | --- | --- | --- |
|  | Kelompok | N | Mean Rank | Sum of Ranks |
| alphamhc | 2 | 5 | 3,20 | 16,00 |
|  | 3 | 5 | 7,80 | 39,00 |
|  | Total | 10 |  |  |

| **Test Statistics^b^** | |
| --- | --- |
|  | alphamhc |
| Mann-Whitney U | 1,000 |
| Wilcoxon W | 16,000 |
| Z | -2,402 |
| Asymp. Sig. (2-tailed) | ,016 |
| Exact Sig. [2*(1-tailed Sig.)] | ,016^a^ |
| a. Not corrected for ties.  b. Grouping Variable: Kelompok | |

| **Ranks** | | | | |
| --- | --- | --- | --- | --- |
|  | Kelompok | N | Mean Rank | Sum of Ranks |
| alphamhc | 2 | 5 | 3,60 | 18,00 |
|  | 4 | 5 | 7,40 | 37,00 |
|  | Total | 10 |  |  |

| **Test Statistics^b^** | |
| --- | --- |
|  | alphamhc |
| Mann-Whitney U | 3,000 |
| Wilcoxon W | 18,000 |
| Z | -1,984 |
| Asymp. Sig. (2-tailed) | ,047 |
| Exact Sig. [2*(1-tailed Sig.)] | ,056^a^ |
| a. Not corrected for ties.  b. Grouping Variable: Kelompok | |

| **Ranks** | | | | |
| --- | --- | --- | --- | --- |
|  | Kelompok | N | Mean Rank | Sum of Ranks |
| alphamhc | 3 | 5 | 5,40 | 27,00 |
|  | 4 | 5 | 5,60 | 28,00 |
|  | Total | 10 |  |  |

| **Test Statistics^b^** | |
| --- | --- |
|  | alphamhc |
| Mann-Whitney U | 12,000 |
| Wilcoxon W | 27,000 |
| Z | -,104 |
| Asymp. Sig. (2-tailed) | ,917 |
| Exact Sig. [2*(1-tailed Sig.)] | 1,000^a^ |
| a. Not corrected for ties.  b. Grouping Variable: Kelompok | |

1. PIK3CA

**Oneway**

| **Descriptives** | | | | | | | | |
| --- | --- | --- | --- | --- | --- | --- | --- | --- |
| PIK3CA | | | | | | | | |
|  | N | Mean | Std. Deviation | Std. Error | 95% Confidence Interval for Mean | | Minimum | Maximum |
|  |  |  |  |  | Lower Bound | Upper Bound |  |  |
| 1 | 5 | 1,28765 | ,100828 | ,045092 | 1,16246 | 1,41285 | 1,117 | 1,371 |
| 2 | 5 | 1,09316 | ,045917 | ,020535 | 1,03614 | 1,15017 | 1,046 | 1,163 |
| 3 | 5 | 1,10500 | ,149690 | ,066943 | ,91914 | 1,29087 | ,872 | 1,271 |
| 4 | 5 | 1,31396 | ,178785 | ,079955 | 1,09197 | 1,53595 | 1,117 | 1,559 |
| Total | 20 | 1,19994 | ,157635 | ,035248 | 1,12617 | 1,27372 | ,872 | 1,559 |

| **Test of Homogeneity of Variances** | | | |
| --- | --- | --- | --- |
| PIK3CA | | | |
| Levene Statistic | df1 | df2 | Sig. |
| 2,707 | 3 | 16 | ,080 |

| **Tests of Normality** | | | | | | | |
| --- | --- | --- | --- | --- | --- | --- | --- |
|  | Kelompok | Kolmogorov-Smirnov^a^ | | | Shapiro-Wilk | | |
|  |  | Statistic | df | Sig. | Statistic | df | Sig. |
| PIK3CA | 1 | ,277 | 5 | ,200^*^ | ,824 | 5 | ,125 |
|  | 2 | ,279 | 5 | ,200^*^ | ,915 | 5 | ,500 |
|  | 3 | ,212 | 5 | ,200^*^ | ,945 | 5 | ,699 |
|  | 4 | ,263 | 5 | ,200^*^ | ,934 | 5 | ,621 |
| a. Lilliefors Significance Correction  *. This is a lower bound of the true significance. | | | | | | | |

| **ANOVA** | | | | | |
| --- | --- | --- | --- | --- | --- |
| PIK3CA | | | | | |
|  | Sum of Squares | df | Mean Square | F | Sig. |
| Between Groups | ,206 | 3 | ,069 | 4,112 | ,024 |
| Within Groups | ,267 | 16 | ,017 |  |  |
| Total | ,472 | 19 |  |  |  |

**Post Hoc Tests**

| **Multiple Comparisons** | | | | | | |
| --- | --- | --- | --- | --- | --- | --- |
| PIK3CA  LSD | | | | | | |
| (I) Kelompok | (J) Kelompok | Mean Difference (I-J) | Std. Error | Sig. | 95% Confidence Interval | |
|  |  |  |  |  | Lower Bound | Upper Bound |
| 1 | 2 | ,194496^*^ | ,081637 | ,030 | ,02143 | ,36756 |
|  | 3 | ,182649^*^ | ,081637 | ,040 | ,00959 | ,35571 |
|  | 4 | -,026307 | ,081637 | ,751 | -,19937 | ,14676 |
| 2 | 1 | -,194496^*^ | ,081637 | ,030 | -,36756 | -,02143 |
|  | 3 | -,011847 | ,081637 | ,886 | -,18491 | ,16122 |
|  | 4 | -,220803^*^ | ,081637 | ,016 | -,39387 | -,04774 |
| 3 | 1 | -,182649^*^ | ,081637 | ,040 | -,35571 | -,00959 |
|  | 2 | ,011847 | ,081637 | ,886 | -,16122 | ,18491 |
|  | 4 | -,208956^*^ | ,081637 | ,021 | -,38202 | -,03589 |
| 4 | 1 | ,026307 | ,081637 | ,751 | -,14676 | ,19937 |
|  | 2 | ,220803^*^ | ,081637 | ,016 | ,04774 | ,39387 |
|  | 3 | ,208956^*^ | ,081637 | ,021 | ,03589 | ,38202 |
| *. The mean difference is significant at the 0.05 level. | | | | | | |

1. mTOR

**Oneway**

| **Descriptives** | | | | | | | | |
| --- | --- | --- | --- | --- | --- | --- | --- | --- |
| mTOR | | | | | | | | |
|  | N | Mean | Std. Deviation | Std. Error | 95% Confidence Interval for Mean | | Minimum | Maximum |
|  |  |  |  |  | Lower Bound | Upper Bound |  |  |
| 1 | 5 | 1,09097 | ,036563 | ,016351 | 1,04557 | 1,13637 | 1,050 | 1,126 |
| 2 | 5 | ,97232 | ,047336 | ,021170 | ,91355 | 1,03110 | ,923 | 1,046 |
| 3 | 5 | ,97069 | ,111068 | ,049671 | ,83279 | 1,10860 | ,807 | 1,066 |
| 4 | 5 | 1,21758 | ,114409 | ,051165 | 1,07552 | 1,35964 | 1,096 | 1,395 |
| Total | 20 | 1,06289 | ,130407 | ,029160 | 1,00186 | 1,12393 | ,807 | 1,395 |

| **Test of Homogeneity of Variances** | | | |
| --- | --- | --- | --- |
| mTOR | | | |
| Levene Statistic | df1 | df2 | Sig. |
| 3,012 | 3 | 16 | ,061 |

| **Tests of Normality** | | | | | | | |
| --- | --- | --- | --- | --- | --- | --- | --- |
|  | Kelompok | Kolmogorov-Smirnov^a^ | | | Shapiro-Wilk | | |
|  |  | Statistic | df | Sig. | Statistic | df | Sig. |
| mTOR | 1 | ,243 | 5 | ,200^*^ | ,838 | 5 | ,161 |
|  | 2 | ,191 | 5 | ,200^*^ | ,939 | 5 | ,661 |
|  | 3 | ,308 | 5 | ,137 | ,857 | 5 | ,218 |
|  | 4 | ,186 | 5 | ,200^*^ | ,949 | 5 | ,729 |
| a. Lilliefors Significance Correction  *. This is a lower bound of the true significance | | | | | | | |

| **ANOVA** | | | | | |
| --- | --- | --- | --- | --- | --- |
| mTOR | | | | | |
|  | Sum of Squares | df | Mean Square | F | Sig. |
| Between Groups | ,207 | 3 | ,069 | 9,521 | ,001 |
| Within Groups | ,116 | 16 | ,007 |  |  |
| Total | ,323 | 19 |  |  |  |

**Post Hoc Tests**

| **Multiple Comparisons** | | | | | | |
| --- | --- | --- | --- | --- | --- | --- |
| mTOR  LSD | | | | | | |
| (I) Kelompok | (J) Kelompok | Mean Difference (I-J) | Std. Error | Sig. | 95% Confidence Interval | |
|  |  |  |  |  | Lower Bound | Upper Bound |
| 1 | 2 | ,118651^*^ | ,053854 | ,043 | ,00448 | ,23282 |
|  | 3 | ,120279^*^ | ,053854 | ,040 | ,00611 | ,23445 |
|  | 4 | -,126609^*^ | ,053854 | ,032 | -,24077 | -,01244 |
| 2 | 1 | -,118651^*^ | ,053854 | ,043 | -,23282 | -,00448 |
|  | 3 | ,001628 | ,053854 | ,976 | -,11254 | ,11579 |
|  | 4 | -,245259^*^ | ,053854 | ,000 | -,35943 | -,13109 |
| 3 | 1 | -,120279^*^ | ,053854 | ,040 | -,23445 | -,00611 |
|  | 2 | -,001628 | ,053854 | ,976 | -,11579 | ,11254 |
|  | 4 | -,246887^*^ | ,053854 | ,000 | -,36105 | -,13272 |
| 4 | 1 | ,126609^*^ | ,053854 | ,032 | ,01244 | ,24077 |
|  | 2 | ,245259^*^ | ,053854 | ,000 | ,13109 | ,35943 |
|  | 3 | ,246887^*^ | ,053854 | ,000 | ,13272 | ,36105 |
| *. The mean difference is significant at the 0.05 level. | | | | | | |

1. LC3

**Oneway**

| **Descriptives** | | | | | | | | |
| --- | --- | --- | --- | --- | --- | --- | --- | --- |
| LC3 | | | | | | | | |
|  | N | Mean | Std. Deviation | Std. Error | 95% Confidence Interval for Mean | | Minimum | Maximum |
|  |  |  |  |  | Lower Bound | Upper Bound |  |  |
| 1 | 5 | 1,24960 | ,098523 | ,044061 | 1,12727 | 1,37193 | 1,120 | 1,396 |
| 2 | 5 | 1,12480 | ,050613 | ,022635 | 1,06196 | 1,18764 | 1,079 | 1,210 |
| 3 | 5 | ,97640 | ,184055 | ,082312 | ,74787 | 1,20493 | ,759 | 1,124 |
| 4 | 5 | ,94640 | ,062983 | ,028167 | ,86820 | 1,02460 | ,869 | 1,031 |
| Total | 20 | 1,07430 | ,161663 | ,036149 | ,99864 | 1,14996 | ,759 | 1,396 |

| **Test of Homogeneity of Variances** | | | |
| --- | --- | --- | --- |
| LC3 | | | |
| Levene Statistic | df1 | df2 | Sig. |
| 7,761 | 3 | 16 | ,002 |

**Kruskal-Wallis Test**

| **Ranks** | | | |
| --- | --- | --- | --- |
|  | Kelompok | N | Mean Rank |
| LC3 | 1 | 5 | 17,40 |
|  | 2 | 5 | 12,00 |
|  | 3 | 5 | 7,60 |
|  | 4 | 5 | 5,00 |
|  | Total | 20 |  |

| **Test Statistics^a,b^** | |
| --- | --- |
|  | LC3 |
| Chi-Square | 12,646 |
| df | 3 |
| Asymp. Sig. | ,005 |
| a. Kruskal Wallis Test  b. Grouping Variable: Kelompok | |

**Mann-Whitney Test**

| **Ranks** | | | | |
| --- | --- | --- | --- | --- |
|  | Kelompok | N | Mean Rank | Sum of Ranks |
| LC3 | 1 | 5 | 7,60 | 38,00 |
|  | 2 | 5 | 3,40 | 17,00 |
|  | Total | 10 |  |  |

| **Test Statistics^b^** | |
| --- | --- |
|  | LC3 |
| Mann-Whitney U | 2,000 |
| Wilcoxon W | 17,000 |
| Z | -2,193 |
| Asymp. Sig. (2-tailed) | ,028 |
| Exact Sig. [2*(1-tailed Sig.)] | ,032^a^ |
| a. Not corrected for ties.  b. Grouping Variable: Kelompok | |

| **Ranks** | | | | |
| --- | --- | --- | --- | --- |
|  | Kelompok | N | Mean Rank | Sum of Ranks |
| LC3 | 1 | 5 | 7,80 | 39,00 |
|  | 3 | 5 | 3,20 | 16,00 |
|  | Total | 10 |  |  |

| **Test Statistics^b^** | |
| --- | --- |
|  | LC3 |
| Mann-Whitney U | 1,000 |
| Wilcoxon W | 16,000 |
| Z | -2,402 |
| Asymp. Sig. (2-tailed) | ,016 |
| Exact Sig. [2*(1-tailed Sig.)] | ,016^a^ |
| a. Not corrected for ties.  b. Grouping Variable: Kelompok | |

| **Ranks** | | | | |
| --- | --- | --- | --- | --- |
|  | Kelompok | N | Mean Rank | Sum of Ranks |
| LC3 | 1 | 5 | 8,00 | 40,00 |
|  | 4 | 5 | 3,00 | 15,00 |
|  | Total | 10 |  |  |

| **Test Statistics^b^** | |
| --- | --- |
|  | LC3 |
| Mann-Whitney U | ,000 |
| Wilcoxon W | 15,000 |
| Z | -2,611 |
| Asymp. Sig. (2-tailed) | ,009 |
| Exact Sig. [2*(1-tailed Sig.)] | ,008^a^ |
| a. Not corrected for ties.  b. Grouping Variable: Kelompok | |

| **Ranks** | | | | |
| --- | --- | --- | --- | --- |
|  | Kelompok | N | Mean Rank | Sum of Ranks |
| LC3 | 2 | 5 | 6,60 | 33,00 |
|  | 3 | 5 | 4,40 | 22,00 |
|  | Total | 10 |  |  |

| **Test Statistics^b^** | |
| --- | --- |
|  | LC3 |
| Mann-Whitney U | 7,000 |
| Wilcoxon W | 22,000 |
| Z | -1,149 |
| Asymp. Sig. (2-tailed) | ,251 |
| Exact Sig. [2*(1-tailed Sig.)] | ,310^a^ |
| a. Not corrected for ties.  b. Grouping Variable: Kelompok | |

| **Ranks** | | | | |
| --- | --- | --- | --- | --- |
|  | Kelompok | N | Mean Rank | Sum of Ranks |
| LC3 | 2 | 5 | 8,00 | 40,00 |
|  | 4 | 5 | 3,00 | 15,00 |
|  | Total | 10 |  |  |

| **Test Statistics^b^** | |
| --- | --- |
|  | LC3 |
| Mann-Whitney U | ,000 |
| Wilcoxon W | 15,000 |
| Z | -2,611 |
| Asymp. Sig. (2-tailed) | ,009 |
| Exact Sig. [2*(1-tailed Sig.)] | ,008^a^ |
| a. Not corrected for ties.  b. Grouping Variable: Kelompok | |

| **Ranks** | | | | |
| --- | --- | --- | --- | --- |
|  | Kelompok | N | Mean Rank | Sum of Ranks |
| LC3 | 3 | 5 | 6,00 | 30,00 |
|  | 4 | 5 | 5,00 | 25,00 |
|  | Total | 10 |  |  |

| **Test Statistics^b^** | |
| --- | --- |
|  | LC3 |
| Mann-Whitney U | 10,000 |
| Wilcoxon W | 25,000 |
| Z | -,522 |
| Asymp. Sig. (2-tailed) | ,602 |
| Exact Sig. [2*(1-tailed Sig.)] | ,690^a^ |
| a. Not corrected for ties.  b. Grouping Variable: Kelompok | |

1. P62

**Oneway**

| **Descriptives** | | | | | | | | |
| --- | --- | --- | --- | --- | --- | --- | --- | --- |
| p62 | | | | | | | | |
|  | N | Mean | Std. Deviation | Std. Error | 95% Confidence Interval for Mean | | Minimum | Maximum |
|  |  |  |  |  | Lower Bound | Upper Bound |  |  |
| 1 | 5 | ,96678 | ,076927 | ,034403 | ,87127 | 1,06230 | ,849 | 1,043 |
| 2 | 5 | ,81850 | ,031058 | ,013890 | ,77994 | ,85707 | ,790 | ,864 |
| 3 | 5 | ,83357 | ,095197 | ,042573 | ,71537 | ,95178 | ,741 | ,977 |
| 4 | 5 | 1,10746 | ,093957 | ,042019 | ,99080 | 1,22413 | ,971 | 1,207 |
| Total | 20 | ,93158 | ,139910 | ,031285 | ,86610 | ,99706 | ,741 | 1,207 |

| **Test of Homogeneity of Variances** | | | |
| --- | --- | --- | --- |
| p62 | | | |
| Levene Statistic | df1 | df2 | Sig. |
| 1,631 | 3 | 16 | ,222 |

| **Tests of Normality** | | | | | | | |
| --- | --- | --- | --- | --- | --- | --- | --- |
|  | Kelompok | Kolmogorov-Smirnov^a^ | | | Shapiro-Wilk | | |
|  |  | Statistic | df | Sig. | Statistic | df | Sig. |
| p62 | 1 | ,252 | 5 | ,200^*^ | ,921 | 5 | ,533 |
|  | 2 | ,294 | 5 | ,183 | ,886 | 5 | ,335 |
|  | 3 | ,186 | 5 | ,200^*^ | ,932 | 5 | ,609 |
|  | 4 | ,188 | 5 | ,200^*^ | ,957 | 5 | ,786 |
| a. Lilliefors Significance Correction  *. This is a lower bound of the true significance. | | | | | | | |

| **ANOVA** | | | | | |
| --- | --- | --- | --- | --- | --- |
| p62 | | | | | |
|  | Sum of Squares | df | Mean Square | F | Sig. |
| Between Groups | ,273 | 3 | ,091 | 14,684 | ,000 |
| Within Groups | ,099 | 16 | ,006 |  |  |
| Total | ,372 | 19 |  |  |  |

**Post Hoc Tests**

| **Multiple Comparisons** | | | | | | |
| --- | --- | --- | --- | --- | --- | --- |
| p62  LSD | | | | | | |
| (I) Kelompok | (J) Kelompok | Mean Difference (I-J) | Std. Error | Sig. | 95% Confidence Interval | |
|  |  |  |  |  | Lower Bound | Upper Bound |
| 1 | 2 | ,148281^*^ | ,049772 | ,009 | ,04277 | ,25379 |
|  | 3 | ,133212^*^ | ,049772 | ,017 | ,02770 | ,23872 |
|  | 4 | -,140678^*^ | ,049772 | ,012 | -,24619 | -,03517 |
| 2 | 1 | -,148281^*^ | ,049772 | ,009 | -,25379 | -,04277 |
|  | 3 | -,015069 | ,049772 | ,766 | -,12058 | ,09044 |
|  | 4 | -,288959^*^ | ,049772 | ,000 | -,39447 | -,18345 |
| 3 | 1 | -,133212^*^ | ,049772 | ,017 | -,23872 | -,02770 |
|  | 2 | ,015069 | ,049772 | ,766 | -,09044 | ,12058 |
|  | 4 | -,273890^*^ | ,049772 | ,000 | -,37940 | -,16838 |
| 4 | 1 | ,140678^*^ | ,049772 | ,012 | ,03517 | ,24619 |
|  | 2 | ,288959^*^ | ,049772 | ,000 | ,18345 | ,39447 |
|  | 3 | ,273890^*^ | ,049772 | ,000 | ,16838 | ,37940 |
| *. The mean difference is significant at the 0.05 level. | | | | | | |
